# In Memory of the Late Honorary Editor-in-Chief, Professor Yukiyasu Sezai

**DOI:** 10.5761/atcs.ob.25-01000

**Published:** 2025-04-05

**Figure:**
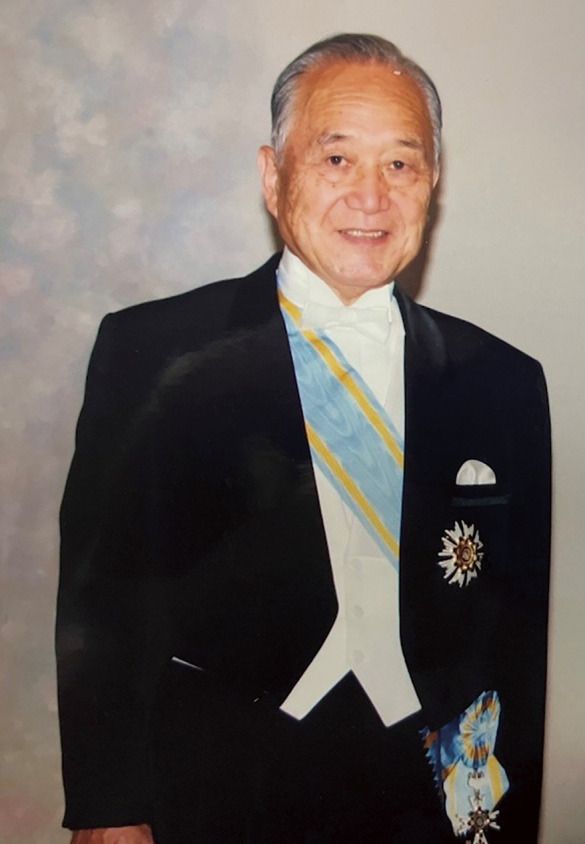


Professor Yukiyasu Sezai, a pioneer of cardiac surgery in Japan and Honorary Editor-in-Chief of *Annals of Thoracic and Cardiovascular Surgery* (ATCS), passed away on February 15, 2025, at the age of 94. We extend our deepest condolences and pray for the repose of his soul.

Professor Sezai graduated from Nihon University School of Medicine in 1955 and came to the United States in 1965 as a Fulbright Scholar to work under Professor Albert Starr at Oregon University, where he was appointed Guest Professor. After returning to Japan, he was appointed Professor of Cardiovascular Surgery at Nihon University in 1975, Dean of Nihon University School of Medicine in 1989, and the 10th President of Nihon University in 1996.

As a cardiac surgeon, he was the first in Japan to successfully perform coronary artery bypass grafting (off-pump) in 1970, valve replacement using St. Jude Medical valves in 1977, and the weaning of ventricular assist devices in 1982.

In 1995, together with Professor Juro Wada, he played a crucial role in launching the English edition of ATCS, significantly contributing to the international development of cardiovascular surgery.

Throughout his distinguished career, Professor Sezai held numerous key positions, including President of the International Rotary Blood Pump Society, President of the Japanese Association for Coronary Artery Surgery, President of the Japanese Society for Artificial Organs, Chairman of the Japan Association of Medical Colleges and University Hospitals, Executive Director of the Federation of Japanese Private Colleges and Universities Associations, and Chairman of the Council for International Educational Exchange.

His outstanding contributions were widely recognized, earning him prestigious awards such as the 5th Mitsukoshi Medical Award in 1977, the Hans Selye Award in 1991, the National University of Jordan Award and the University of Prague Award in 1994, and the University Award from the University of Giessen in 1995. Furthermore, he was awarded the “Palmes académiques (Commandeur)” by the French government, the Order of Friendship of the Russian Federation from President Gorbachev, and the “Grand Cordon of the Order of the Sacred Treasure” from Emperor Akihito of Japan in 2006.

Professor Sezai’s passing is an immense loss to the medical community, and his remarkable achievements will be remembered forever.

Yoshinori Watanabe, MD. PhD.

*Editor-in-Chief, Annals of Thoracic and Cardiovascular Surgery*

*President, Toho University*

